# Systematical Characterization of the Cotton *Di19* Gene Family and the Role of *GhDi19-3* and *GhDi19-4* as Two Negative Regulators in Response to Salt Stress

**DOI:** 10.3390/antiox11112225

**Published:** 2022-11-11

**Authors:** Lanjie Zhao, Youzhong Li, Yan Li, Wei Chen, Jinbo Yao, Shengtao Fang, Youjun Lv, Yongshan Zhang, Shouhong Zhu

**Affiliations:** 1 State Key Laboratory of Cotton Biology, Institute of Cotton Research, Chinese Academy of Agricultural Sciences, Anyang 455000, China; 2 Xinjiang Production & Construction Group Key Laboratory of Crop Germplasm Enhancement and Gene Resources Utilization, Biotechnology Research Institute, Xinjiang Academy of Agricultural and Reclamation Science, Shihezi 832000, China; 3 Anyang Institute of Technology, Anyang 455000, China

**Keywords:** cotton, Di19, zinc-finger protein, ROS, abiotic stress

## Abstract

Drought-induced 19 (Di19) protein is a Cys2/His2 (C2H2) type zinc-finger protein, which plays a crucial role in plant development and in response to abiotic stress. This study systematically investigated the characteristics of the *GhDi19* gene family, including the member number, gene structure, chromosomal distribution, promoter cis-elements, and expression profiles. Transcriptomic analysis indicated that some *GhDi19s* were up-regulated under heat and salt stress. Particularly, two nuclear localized proteins, *GhDi19-3* and *GhDi19-4*, were identified as being in potential salt stress responsive roles. *GhDi19-3* and *GhDi19-4* decreased sensitivity under salt stress through virus-induced gene silencing (VIGS), and showed significantly lower levels of H_2_O_2_, malondialdehyde (MDA), and peroxidase (POD) as well as significantly increased superoxide dismutase (SOD) activity. This suggested that their abilities were improved to effectively reduce the reactive oxygen species (ROS) damage. Furthermore, certain calcium signaling and abscisic acid (ABA)-responsive gene expression levels showed up- and down-regulation changes in target gene-silenced plants, suggesting that *GhDi19-3* and *GhDi19-4* were involved in calcium signaling and ABA signaling pathways in response to salt stress. In conclusion, GhDi19-3 and GhDi19-4, two negative transcription factors, were found to be responsive to salt stress through calcium signaling and ABA signaling pathways.

## 1. Introduction

Adverse abiotic environmental factors, including drought, soil salinity, and heat and cold during crop development, limit the production area of crops and adversely affect both crop productivity and quality [1]. Hence, investigating the mechanism of plants adaptation toward stress cues and strategies to cope with adverse environmental factors is critical for maintaining crop production and food security [2]. Plants have developed and formed various levels of stress response mechanism and physiological mechanisms as well as other adaptive strategies to deal with abiotic environmental threats and relay this information to trigger appropriate physiological and cellular responses. At present, there have been two types of proteins reported to participate in the regulatory process of stress conditions [3]. The first type is the functional proteins involved in the direct regulation of stress, including osmotic regulators, ion channel proteins, antioxidant protection enzymes, etc., and the second type is the regulatory proteins, including transcription factors, plant protein phosphatases, and other signaling proteins. Transcription factors, including TCP [4], NAC [5], and C2H2 type zinc-finger proteins [6,7], are identified in varied abiotic stress signals and hormone regulatory pathways. Di19 proteins involved in plant abiotic stress belong to a small zinc-finger protein transcription factor gene family in plants. Di19 proteins are comprised two unusual, conserved domains, the Di19_zinc-binding domain and the Di19_C terminal domain [8]. Di19 proteins have been reported in *Arabidopsis* [9], cotton [10], rice [8], soybean [11], maize [12], moso bamboo [13], and other species.

At present, *Arabidopsis* is the most indepth studied plant of the *Di19* gene family [14]. Previous studies have revealed that there are seven *AtDi19* genes (*AtDi19-1*-to *AtDi19-7*) in the model plant *Arabidopsis*, which could be induced to express under stress. For example, the expression of *AtDi19-1* and *AtDi19-3* is regulated under drought stress, while the expression of *AtDi19-2* and *AtDi19-4* is regulated by high salt stress [8,14]. A previous study showed that the *Atdi19-1* mutant exhibited a hypersensitive phenotype under drought stress [15]. *AtDi19-1* up-regulated the expression level of pathogenesis-related (*PR1*, *PR2*, and *PR5*) genes through binding to the TACA (A/G) T element in *PR1*, *PR2*, and *PR5* gene promoters under drought stress [15]. While AtDi19-3 has also been able to bind to the TACA (A/G) T element and the loss of the *AtDi19-3* function in plants has led to an enhanced resistance to drought, salinity, and ABA stress. Compared to the wild-type, *Atdi19-3*-overexpressed plants have been shown to be more sensitive to drought, salinity, and ABA [16]. Most *AtDi19* gene family members have been shown to interact with and be phosphorylated by calcium signal-related protein kinases to perform their functions [8]. In vitro, AtDi19-1 has interacted with AtCPK11/CDPK2 [17], and its transactivation activity was enhanced by AtCPK11/CDPK2 [15]. AtDi19-2 can be strongly phosphorylated by AtCPK16 [18]. AtDi19-3 has interacted with and was phosphorylated by the calcineurin B-like interacting protein kinase 11 (CIPK11) protein, which partially mediated in drought stress response through the regulating of *AtDi19-3* [19]. In addition, Di19-3 has been shown to interact with AtIAA14, which affects the lateral root development in *Arabidopsis* [20]. *AtDi19-7* (also known as *HRB1*) expression level was mediated by light signal and was involved in the red-light response mediated by phytochrome B and the blue-light response mediated by cryptochrome [21]. AtDi19-7 protein also interacts with the F-box protein LKP2 in the light signaling pathway [22]. In short, Di19 protein has been demonstrated to have important functions in plant development and abiotic stress response.

Di19 proteins also play an unusual role in coping with adversity in important food and cash crops. Previous studies have shown that overexpression of the *TaDi19A* gene in *Arabidopsis* plant leads to the reduced tolerance to salt stress, ABA, and mannitol [23]. *TaDi19A* gene transcription also regulates ABA and SOS signaling networks in stress response [23]. *OsDi19-3* and *OsDi19-4* gene expression levels are induced by dehydration and salt stress in rice [8]. Overexpression of *OsDi19-4* has enhanced the scavenging activity of reactive oxygen species (ROS) and has improved the ability of rice to resist drought stress, whereas, the *OsDi19-4* knockout lines have been found to be less sensitive to ABA treatment [8,24]. Interaction between OsCDPK14 and OsDi19-4, was found to be responsible for the phosphorylation of OsDi19-4, which was further improved after ABA treatment [24]. Further, OsDi19-4 was shown to directly bind to two ABA-responsive gene promoters, namely *OsASPG1* and *OsNAC18*, and to participate in their expression [24]. This suggested that OsDi19-4 functions downstream of OsCDPK14 to positively promote ABA responses through regulating ABA-responsive gene expression in rice [24]. Additionally, ABA-induced OsDi19-1, together with OsSCP46 and other unknown proteins, function as a protein complex in the proteolysis during seed development [25]. *ZmDi19-1* expression is also induced by various abiotic stresses in maize and has been shown to recognize and bind to the TACA (A/G) T element [12]. Ectopic expression of *ZmDi19-1* in *Arabidopsis* affected downstream stress-related gene expression and improved its salt tolerance [12]. Soybean GmDi19-5 interacts with GmLEA3.1 and the sensitivity to abiotic stress of its transgenic plants were improved, where GmDi19-5 acted as a negative regulator in abiotic stress response through ABA and SOS signaling pathways [11]. In addition, PheDi19-8 protein interacts with the PheCDPK22 protein and has also been shown to play a role in drought stress response in moso bamboo [13]. These results suggest that different Di19 proteins perform different functions when plants are responding to abiotic stress.

In addition to being an important natural fiber material for the textile industry, cotton is also a pioneer crop in the restoration of saline-alkali soil. A previous study has indicated that the *GhDi19-1* and *GhDi19-2* up-regulated expressions in cotton are induced by salt and drought stress [10]. Although ectopic expression of *GhDi19-1* and *GhDi19-2* in *Arabidopsis* have conferred high sensitivity to salt and ABA [10,26], the mechanisms underlying the involvement of GhDi19s in stress responses have remained unknown. Due to the pivotal roles of Di19 proteins in plant stress responses, the Di19 gene family in cotton and the functions and regulatory networks of *Di19-3* and *Di19-4* genes in abiotic stress are systematically investigated in this study. Our investigation provides a theoretical basis for understanding the regulatory mechanism of Di19s in cotton under salt stress and enriches the genetic resources for the breeding of stress resistance cotton.

## 2. Materials and Methods

### 2.1. Plant Materials and Growth Conditions

Upland cotton (*Gossypium hirsutum* cv. Zhongmiansuo100) seeds were soaked in warm sterile water for 12 h after removing the lint using sulfuric acid, and then were sown on the germination plate and incubated at 28 °C for 3 days. Seedlings were transferred to small pots of the nutrient soil mixed with vermiculite (the ratio of nutrient soil and vermiculite was 3:1 (V:V)) and were grown in the greenhouse (at 23 °C, 16 h light/8 h dark cycle) of the Cotton Research Institute of the Chinese Academy of Agricultural Sciences (China, Anyang). Three-week-old tobacco (*N. benthamiana*) seedlings from the same growth conditions of 23 °C, 16 h light/8 h dark cycle were used in the subcellular localization analysis of target genes.

### 2.2. Sequence Identification and Phylogenetic Analysis

Genome data of *G. hirsutum* (ZJU, version 2.0), *G. arboreum* (CRI, version 1.0), and *G. raimondii* (JGI, version 2.0) were downloaded from COTTONGEN (http://www.cottongen.org, accessed on 3 September 2021) [27] and the Cotton Functional Genomics Database (CottonFGD) (https://cottonfgd.net/, accessed on 6 September 2021) [28], while the sequence of reported AtDi19 proteins were downloaded from The Arabidopsis Information Resource (TAIR, version 10, http://www.arabidopsis.org, accessed on 6 September 2021) [14]. The *Di19* genome data of *Theobroma cacao* (version 2.1), *Vitis vinifera* (Version 2.1), *Glycine max* (version 2.0), *Populus trichocarpa* (version 4.1), *Zea mays* (version 1.1), *Oryza sativa* (version 7.0), *Sorghum bicolor* (Version 3.1), and *G. hirsutum* (JGI, version 2.0) were retrieved from Phytozome (https://phytozome.jgi.doe.gov/pz/portal.html, accessed on 16 November 2021) [29].

Using the BLASTP program, amino acid sequences of AtDi19s were used as a query sequence, and genes with the e-values less than 1e^−5^ were selected in the protein database of the three cotton species and other plant species as candidate genes. Interproscan 5 (http://www.ebi.ac.uk/interpro/, accessed on 17 December 2021) [30] and SMART (http://smart.embl.de/, accessed on 17 December 2021) online tools [31] were used, the Di19_zinc-binding domain (IPR008598) and the Di19_C terminal domain (IPR027935) were retrieved and used as the query sequences [9,10], and ultimately *Di19* genes were identified in ten plant species. Sequence alignment was performed using DNAMAN while phylogenetic trees were generated using MEGA 7.0 [32].

### 2.3. Sequence Characteristics and Chromosome Distribution of Di19s

Molecular weights and theoretical isoelectric points (pIs) of Di19 proteins were analyzed using ExPASy (http://web.expasy.org/protparam/, accessed on 27 December 2021). Prediction of subcellular localization was conducted using the CELLO v.2.5 server [33]. Protein 3D structure of GhDi19-3 and GhDi19-4 was predicted by the SWISS-MODEL server (http://swissmodel.expasy.org, accessed on 16 February 2022) [34]. MapChart software [35] was used to map the *Di19* genes and the cotton chromosomes and GSDS software was used to visualize the exons and introns of *GhDi19s* [36]. The PlantCARE website (http://bioinformatics.psb.ugent.be/webtools/plantcare/html/, accessed on 9 December 2021) was used to predict the cis-elements in the promoter regions (2000 bp) of Di19 genes. Conserved motif and domain were examined using the MEME Suite [37] and TBtools software [38].

### 2.4. Transcriptomic Data and Expression Patterns Analysis of Di19s

*G. hirsutum* ‘TM-1’ transcriptomic data of different tissues and various abiotic stresses were downloaded from COTTONOMICS (http://cotton.zju.edu.cn/, accessed on 20 November 2021) [39]. Heatmaps were generated using expression data by HemI v.1.0 software [40].

The extraction of total RNA and the reverse transcription were prepared according to the previous methods [41]. qRT-PCR primers for *GhDi19s* and the associated network-related genes were shown in Table S1. *GhUBQ7* was selected as an internal reference gene. qRT-PCR was run on a Bio-Rad 7500 fast fluorescence quantitative PCR platform following the operation protocol. Each treatment had three biological replicates, and the relative expression levels of the genes were analyzed using the 2^−ΔΔCt^ method [42].

### 2.5. Protein Association Network of GhDi19s

STRING software (https://string-db.org/, accessed on 24 February 2022) [41] was used to illustrate the association network of GhDi19 proteins based on the orthologs in *Arabidopsis* and *G. raimondii* with a high confidence parameter set at 0.7 and 0.4 thresholds, respectively.

### 2.6. Subcellular Localization of GhDi19-3 and GhDi19-4 in Tobacco

To determine the subcellular localization of GhDi19-3 and GhDi19-4, CDS of *GhDi19-3* and *GhDi19-4* were cloned into P-super1300 vector to generate C-terminally fused green fluorescent protein (GFP) constructs 35S:: GhDi19-3-GFP and 35S:: GhDi19-4-GFP, respectively, using a one-step cloning kit (Vazyme Biotech Ltd., Nanjing, China). Vector construction primers are listed in Table S1. These vectors were firstly transformed into the *Agrobacterium tumefaciens* strain GV3101 and were then infiltrated into the tobacco leaves. After two days, the green fluorescence of the leaf cells was detected under a confocal microscope (Carl Zeiss LSM710, Oberkochen, Germany).

### 2.7. VIGS Vector Construction and Treatments

The VIGS system was generated following the protocol of Shaban et al. [43]. The 213 bp and 396 bp sequence fragments of *GhDi19-3* and *GhDi19-4* were cloned from *G. hirsutum* cv. Zhongmiansuo 100, respectively, and were then cloned into the TRV: 00 plasmids, to generate the TRV: *GhDi19-3* and TRV: *GhDi19-4* vectors using the one-step cloning kit (Vazyme Biotech Ltd., Nanjing, China). TRV: *GhDi19-3*, TRV: *GhDi19-4*, and TRV1 constructs were transformed into the *A. tumefaciens* strain GV3101 by electroporation. Primers for vector construction were listed in Table S1. When *GhCLA1* gene-silenced (positive control) plants showed the albinism phenotype, it indicated that the silencing mechanism had been successfully induced in the positive control. Then the VIGS efficiency of *GhDi19-3* and *GhDi19-4* was verified by qRT-PCR. TRV: 00, TRV: GhDi19-3, and TRV: GhDi19-4, plants were subjected to mock treatment (water) and 400 mM NaCl was used as salt stress treatments for up to ten days. The experiment was repeated three times, and forty plants were used for each treatment.

### 2.8. Detection and Analysis of Salt Stress-Related Physiological Parameters

Physiological parameters, including malondialdehyde (MDA) content, superoxide dismutase (SOD), peroxidase (POD), and H_2_O_2_ activities, were detected in the control and *GhDi19-3*- and *GhDi19-4*-silenced cotton plants under salt treatment. The H_2_O_2_ content was estimated using the protocol of the H_2_O_2_ quantification assay kit (Solarbio, Beijing, China). About 0.1 g of the leaf was ground and blended in a 1 mL lysate. Then the homogenate was centrifuged at 4 °C for 10 min at 8000× *g*. The collected supernatant was collected and then mixed with the same amount of H_2_O_2_ detection reagent and then homogenized by vortexing. Absorbance was measured at 415 nm, and then using the standard curve as the reference to calculate the content of H_2_O_2_. The MDA content, POD, and SOD activities were quantified from 0.1 g cotton leaves using MDA, POD, and SOD assay kits from Solarbio Science and Technology Co., Ltd., Beijing, China. Tissue extracts were prepared according to the manufacturer’s instructions in the relevant kits, as previously described [43].

## 3. Results

### 3.1. Identification and Characterization of Di19 Genes

A total of 16, 8, 8, 5, 11, 6, 7, 7, 7, and 7 *Di19s* were identified from 10 plant species, including *G. hirsutum* (*Gh*), *G. arboreum* (*Ga*), *G. raimondii* (*Gr*), *T. cacao* (*Tc*), *G. max* (*Gm*), *V. vinifera* (*Vv*), *P. trichocarpa* (*Pt*), *S. bicolor* (*Sb*), *Z. mays* (*Zm*), and *O. sativa* (*Os*), respectively (Table S2). In previous studies, *GhDi19-1* (GenBank: GU292049) and *GhDi19-2* (GenBank: GU292050), were cloned from the cDNA library of cotton seedlings [10,26]. In our study, the amino acid sequences of GhDi19-1 and GhDi19-2 were used to run multiple sequence alignments with 16 GhDi19 proteins, respectively. GhDi19-1 had the highest amino acid identity of 49% and 88% with GH_D13G1997.1 and GH_A13G2036.1, respectively, while GhDi19-2 had the highest amino acid identity of 100% and 98% with GH_A11G3034.1 and GH_D11G3065.1, respectively. Based on the sequenced *G. hirsutum* genomes (JGI version) [44] from Phytozome, the GhDi19-1 protein showed 100% and 98% of amino acid sequence identity with Gohir.D13G180800.2 and Gohir.A13G174900.1, respectively, while the GhDi19-2 protein showed 100% and 98% amino acid sequence identity with Gohir.A11G271200.1 and Gohir.D11G281400.1, respectively. These results indicated that *GhDi19-1* and *Gohir.D13G180800.2* were the same gene and that *GhDi19-2*, *GH_A11G3034.1*, and *Gohir.A11G271200.1* were also the same gene. Comparing the information of these genes from two *G. hirsutum* genomes (ZJU version and JGI version), the annotations of *GH_D13G1997.1* and *GH_A13G2036.1* from the *G. hirsutum* genome (ZJU version) was not accurate. Further, using the same method, other Di19 genes were also identified and found that *GH_A11G1176.1*, *GH_D11G1206.1*, *GH_A13G1319.1*, and *GH_D13G1242.1* also had incorrect annotations. Therefore, we corrected the annotation information, as shown in Table S2 and Figure S1. These genes were named *GhDi19-1*, *GhDi19-2*, *GhDi19-3*, and *GhDi19-4*, respectively (Table S2). Other *GhDi19s* were named according to the order of the chromosome localization. The name of *Di19* genes in other plant species also followed the same method.

General biochemical and physical characteristics of *Di19* gene in cotton and other plant species are given in Table S2. The size of GhDi19 proteins ranged from 210 to 239 amino acids, with theoretical pIs ranges from 4.47 to 6.23, and molecular weights ranges from 23.86 to 27.02 kDa (Table S2). Analysis of subcellular localization predicted that 16 GhDi19s were localized in the nucleus (Table S2).

### 3.2. Phylogenetics, Sequence Structure, and Conserved Motif of the Di19s

**Figure 1:**
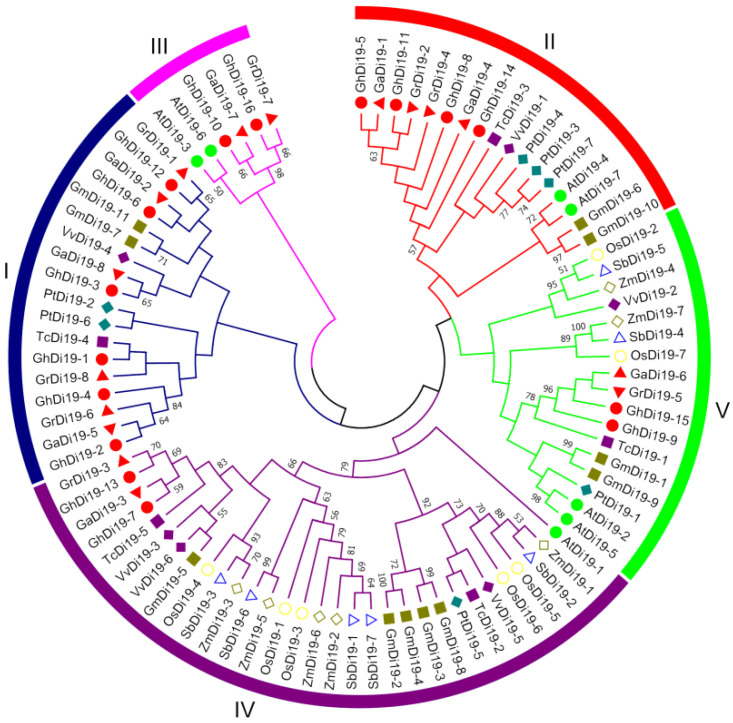
Phylogenic analysis of Di19 proteins in cotton and other plants. The neighbor-joining (NJ) phylogenetic tree was built with MEGA 7.0, and the bootstrap values from 1000 replicates are indicated at each branch. Each plant species was marked with different characters and colors. Di19 proteins of 11 plants were labeled with different colors and patterns.

To investigate the evolutionary relationships among Di19 proteins, 89 Di19 proteins from 11 plant species were analyzed and a phylogenetic tree was constructed (Figure 1, Table S2). These proteins were divided into five different subfamilies, named as I, II, III, IV, and V (Figure 1). In subgroups I, II and III, the Di19 protein from dicotyledonous plants was clustered, while the other two subgroups (IV and V) contained Di19 proteins from both dicotyledonous and monocotyledonous plants (Figure 1). The Di19 proteins in subfamily III had the least number, with only six proteins, while the subgroup IV was the largest, with 31 Di19 proteins (Figure 1). Furthermore, phylogenetic analysis showed that upland cotton had undergone a significant gene expansion due to the presence of almost more than double the number of *Di19* genes compared with all other plant species except for *G. max*. These results indicated that the Di19 proteins were conserved during species evolution.

**Figure 2:**
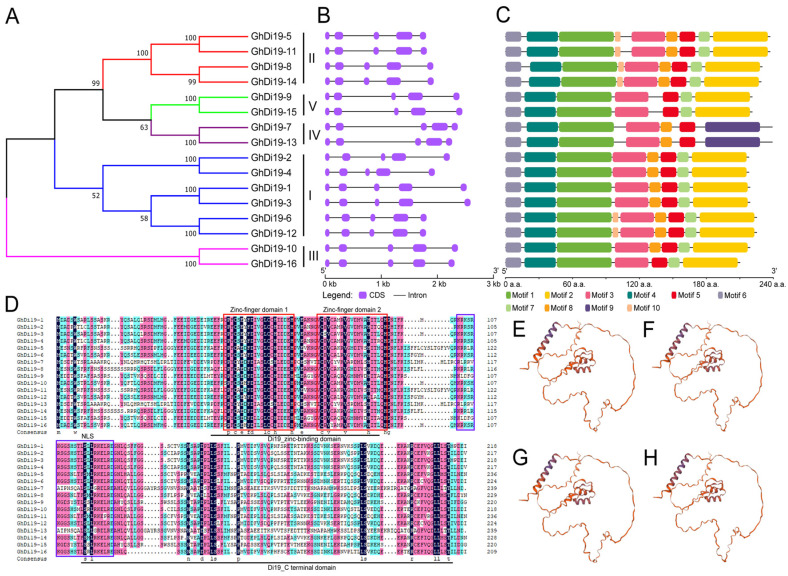
Gene structure, motifs, domains, and protein 3D-structures analysis of 16 *GhDi19* genes in *G. hirsutum*. (**A**) Phylogenetic tree of GhDi19s. A neighbor-joining (NJ) phylogenetic tree was built using MEGA 7.0, and the bootstrap values of 1000 replicates were listed at each branch. (**B**) Structural analysis of 16 *GhDi19s*. (**C**) Motif of 16 GhDi19 proteins. Ten motifs were investigated using the MEME online tool. (**D**) Multiple sequence alignments of 16 GhDi19 proteins. Two zinc-finger domains and one NLS are marked by red and blue boxes, respectively. Di19_zinc-binding domain and the Di19_C terminal domain of Di19 proteins are underlined in black. (**E**–**H**) 3D structures of GhDi19-1, GhDi19-2, GhDi19-3, and GhDi19-4 proteins.

To further investigate the sequences of *GhDi19* genes, the exon and intron structure of *GhDi19* genes (Figure 2) was investigated. It was found that all 16 *GhDi19* members shared similar structures with five exons and four introns (Figure 2B). Based on the results of a motif analysis of GhDi19 proteins using MEME suite, a total of ten conserved motifs were identified. As expected, the GhDi19 proteins from the same subgroup had a similar conserved motif distribution pattern, while the pattern differed between different subgroups (Figure 2A,C). Subgroup V (GhDi19-9 and GhDi19-15) and subgroup IV (GhDi19-7 and GhDi19-13) contained the least motifs (only seven motifs), while subgroup II (GhDi19-5, GhDi19-8, GhDi19-11, and GhDi19-14) contained the most motifs (nine motifs) (Figure 2C). In subgroup I, GhDi19-1, GhDi19-2, GhDi19-3, and GhDi19-4 had only one motif (Motif 10) less compared with GhDI19-6 and GhDi19-12, indicating that these conserved motifs may specify the unique functions of different subgroups of Di19. In general, these conserved structures and motifs reflected the phylogenetic tree analysis results. Further, conserved domains of GhDi19s were demonstrated by multiple protein sequence alignment of GhDi19s. Almost all proteins contained the Di19_zinc-binding domain (IPR008598) and the Di19_C terminal domain (IPR027935) (Figure 2D). The Di19_zinc-binding domain composed of two unusual strictly conserved Cys2/His2 zinc-finger domains (Figure 2D). Additionally, all GhDi19 proteins had the peptide of nuclear localization signal (NLS) (Figure 2D). GhDi19-1, GhDi19-2, GhDi19-3, and GhDi19-4 had the highest protein identities of 63.68%, 62.33%, 64.13%, and 61.43%, respectively, with AtDi19-3 among the seven *AtDi19* genes. Further analysis of the 3D structure of those four GhDi19 proteins using the SWISS-MODEL [34] showed that they had highly similar structures to AtDi19-3 (Figure 2E–H) suggesting potential function similarity.

### 3.3. Conserved Cis-Elements and Expression Profiles of GhDi19 Genes

**Figure 3:**
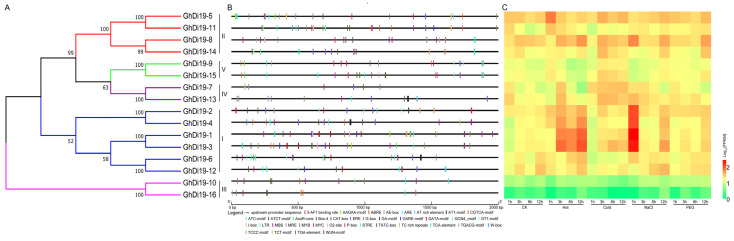
Conserved promoter cis-element and expression profile analysis of *GhDi19s*. (**A**) Phylogenetic tree of GhDi19s. A neighbor-joining (NJ) phylogenetic tree was built with MEGA 7.0, and the bootstrap values from 1000 replicates are indicated at each branch. (**B**) Conserved promoter analysis of GhDi19 genes. Different cis-elements in the 2000bp promoter region upstream of Di19 gene are marked with different color boxes. (**C**) RNA-sequence data analysis of *GhDi19s* under different abiotic stresses (cold, hot, PEG, and NaCl). Transcriptomic data normalization and visualization was performed. The color scale in the lower right corner of the heatmap represents the fragments per kilobase million (FPKM) values, which were standardized by log10.

Cis-elements related to plant growth and development, abiotic or biotic stress response, and phytohormone responses were found in this study (Figure 3). Among the cis-elements related to plant growth and development, the number of light-responsive elements were the majority, including Box-4, as well as certain cis-elements involved in zein metabolism regulation, cis-elements related to meristem expression, cis-elements involved in endosperm expression, and so on (Figure 3, Table S3). Among the cis-elements related to phytohormone response, those related to ABA (such as ABRE and AAGAA-motif), auxin (such as AuxR-core, MYB, and TGA-element), gibberellins (such as GARE-motif and TATC-box), ethylene (such as ERE), MeJA (such as TGACG-motif and CGTCA-motif), and salicylic acid (such as TCA-element) were mainly included. Among them, the number of auxin-responsive elements (such as MYB) was the largest suggesting that *GhDi19* genes may also have important functions in the auxin signal pathway. Further, the number of ARE, MYC, and STRE cis-elements were the largest of the abiotic stress-related cis-elements of *GhDi19s* (Table S3), indicating that certain *GhDi19* genes participated in the stress response, which is consistent with previous studies [10,14,26].

To understand the role of cotton Di19 proteins under abiotic stress, the transcriptome data of 16 *GhDi19s* under different stress conditions, including hot, cold, NaCl, and polyethylene glycol (PEG), were analyzed (Figure 3, Table S4). Compared to the control, the expression of *GhDi19-1*, *GhDi19-2*, *GhDi19-3*, and *GhDi19-4* genes increased significantly under heat stress conditions, reaching the highest level after 3–12 h. Under cold stress conditions, expression levels of *GhDi19-1*, *GhDi19-3*, *GhDi19-7*, *GhDi19-13*, *GhDi19-9*, and *GhDi19-15* were significantly increased compared to the control. These results showed that *GhDi19s* respond to temperature stress. Under salt stress conditions, the expression levels of *GhDi19-1*, *GhDi19-2*, *GhDi19-3*, and *GhDi19-4* genes showed a drastic increase of expression at 1 h after salt stress and reduced afterwards. This suggested that these *GhDi19* genes were related in response to salt stress. However, under drought treatments, most *GhDi19s* expression had no significant changes compared with the control. Based on these results, it was concluded that the *GhDi19s* were differentially regulated under abiotic stress (hot and salt stress) in cotton.

In addition, the transcriptome of *GhDi19* genes were analyzed in different tissues (Figure S2, Table S5). Most *GhDi19* genes exhibited a constitutive expression pattern. *GhDi19-1*, *GhDi19-2*, *GhDi19-3*, and *GhDi19-4* genes were mainly expressed in the ovules (0, 1, and 3 days post anthesis (DPA)) and the fiber tissue at the elongation stage (10, 20, and 25 DPA), while *GhDi19-10* was predominantly expressed in the fiber tissue of 10 DPA. It was speculated that these *GhDi19s* were involved in the ovule’s development and fiber elongation, especially the *GhDi19-10* which possibly was a candidate gene for regulation of fiber elongation in cotton.

Based on the above results, we demonstrated that *GhDi19-1*, *GhDi19-2*, *GhDi19-3*, and *GhDi19-4*, had important functions in cotton, either in response to stress conditions or to regulate the development of cotton fiber (Figure 3 and Figure S2). However, the function of the two genes *GhDi19-1* and *GhDi19-2* has been relatively well documented [10,26]. Therefore, we focused on the other two, *GhDi19-3* and *GhDi19-4*, which were used as candidate genes for further functional verification of their potential roles.

### 3.4. Protein Association Network

**Figure 4:**
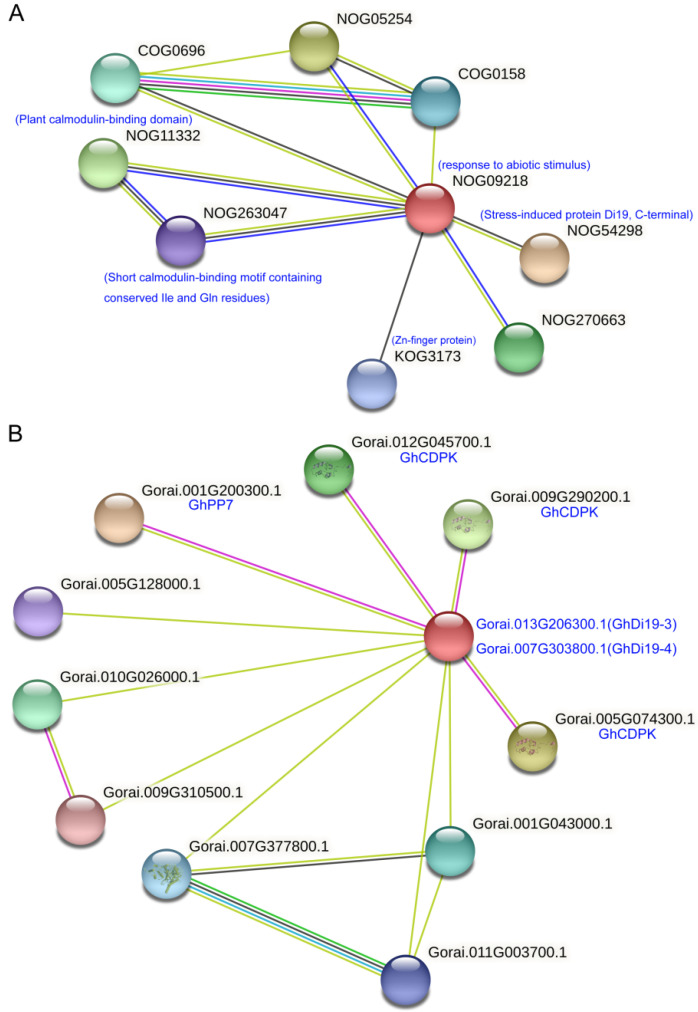
Association network of Di19s. (**A**) Association network of Di19 proteins. A total of nine association protein signaling pathways were identified. The blue letters represent Di19 protein signaling pathway or other protein families. (**B**) Association network of GhDi19-3 and GhDi19-4 with other Di19 proteins. A total of ten association proteins were identified. The blue letters represent CDPK proteins and other proteins.

Based on the association network of Di19s, the functions of cotton *Di19* genes were speculated upon (Figure 4). Overall, we found that the response to the abiotic stimulus signaling pathway (NOG09218) in the network center (Figure 4A) and other signaling pathways related to abiotic stress, including the stress-induced protein Di19, C-terminal (NOG54298) and Zn-finger protein (KOG3173) pathways [8,14], suggesting a certain connection between the Di19 members and the abiotic stress signaling pathway. Plant calmodulin-binding domain (NOG11332) and short calmodulin-binding motif with conserved IIe and Gln residues (NOG263047) association pathways [8,14,18,19] involved in response to abiotic stimuli were found in the association network, indicating that certain Di19 members were also regulated in the abiotic stress response through interaction with the CDPK proteins. Interestingly, through a multiple sequences search, it was found that GhDi19-3 and GhDi19-4 were associated with the CDPK proteins (Gorai.001G200300.1, Gorai.005G074300.1, Gorai.009G290200.1, and Gorai.012G045700.1) (Figure 4B), which were also believed to be special regulators of the plant hormone ABA signaling pathway and that CDPKs performed important functions in response to ABA through triggering downstream related factors [8,14,18,19,45]. Further, GhDi19-3 and GhDi19-4 associated with GhPP7 (Gorai.001G200300.1) (Figure 4B), were thought to be participate in plant photoreceptor regulation that regulated photomorphogenic development.

### 3.5. Subcellular Localization of GhDi19-3 and GhDi19-4 Proteins

**Figure 5:**
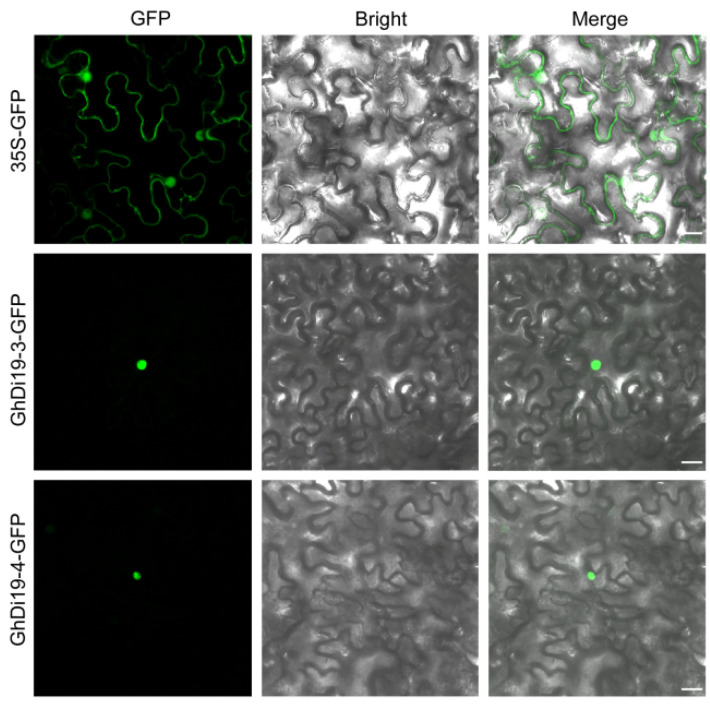
Subcellular localization of GhDi19-3 and GhDi19-4 in tobacco epidermal cells. 35S-GFP, GhDi19-3-GFP, and GhDi19-4-GFP represented confocal imaging of epidermal cells of transiently expression of the control GFP, GhDi19-3 and GhDi19-4, respectively. GFP fluorescence signals were mainly detected in nucleus of the tobacco epidermal cells. GFP, GFP fluorescence images; Bright, bright field image of the same leaf on the left; Merged, GFP fluorescence and bright merged image of the same leaf. Scale bars are 25 µm.

Through the tobacco transient expression system, it was shown that the GhDi19-3 and GhDi19-4 proteins were exclusively localized in the nucleus, whereas the GFP signal of the control expression vector was found in both the nucleus and the plasma membrane (Figure 5). This result together with the presence of the conserved NLS domain in GhDi19-3 and GhDi19-4 proteins (Figure 2D) suggested that GhDi19-3 and GhDi19-4 were nuclear proteins.

### 3.6. Silencing of GhDi19-3 and GhDi19-4 Increases the Tolerance of Cotton to Salt Stress

**Figure 6:**
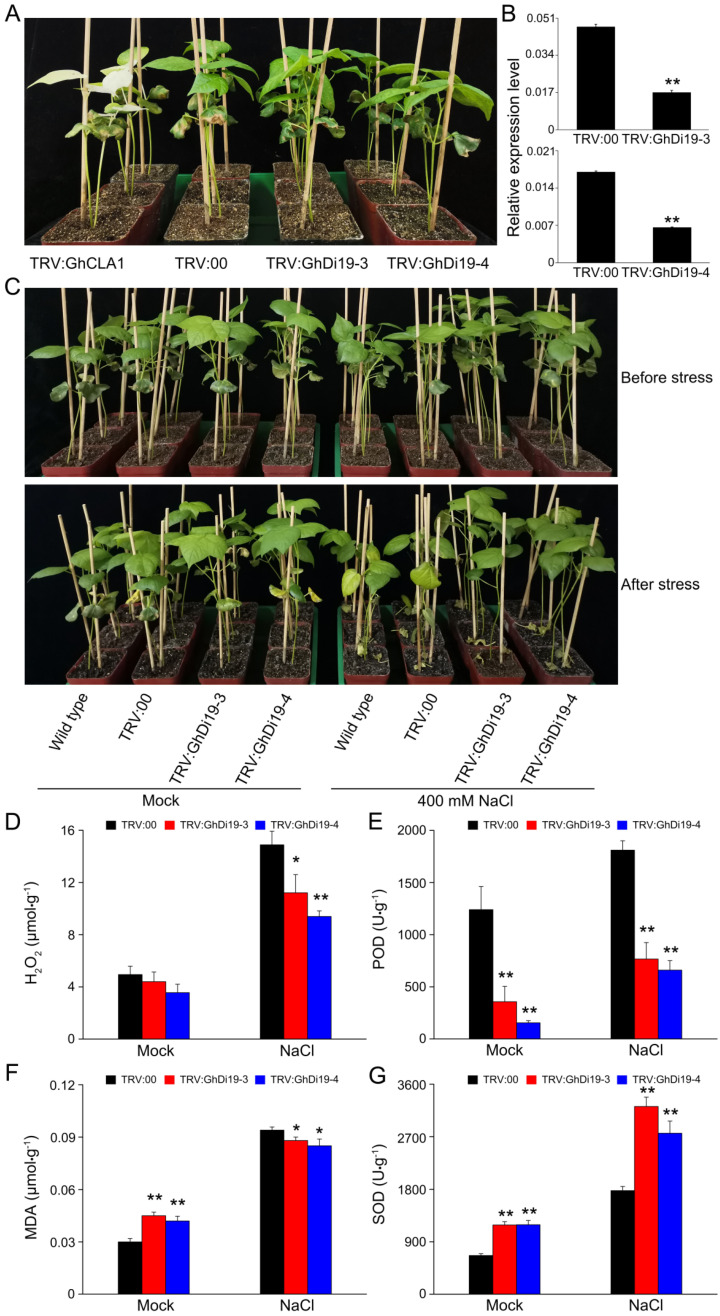
Silenced *GhDi19-3* and *GhDi19-4* improves the tolerance of cotton to salt stress. (**A**) The *GhDi19-3* and *GhDi19-4* silenced genes, control (TRV: 00) and positive control (TRV: GhCLA1) cotton plants before treatments. (**B**) Expression of *GhDi19-3* and *GhDi19-4* in TRV: 00, TRV: GhDi19-3, and TRV: GhDi19-4 cotton plants. (**C**) Phenotype of TRV: 00, TRV: GhDi19-3, and TRV: GhDi19-4 cotton plants under mock and treatment of 400 mM NaCl, H_2_O_2_ contents (**D**), POD (**E**), MDA contents (**F**) and SOD activity (**G**) of the silenced genes and control plants (TRV: 00) under treatments. Asterisks indicate significant differences (independent *t*-tests): * *p* < 0.05; ** *p* < 0.01.

Based on the *GhDi19s* promoter analysis and transcriptome data analysis in different abiotic stressors, it was believed that *GhDi19s* had a potential role in stress responses. Using the VIGS system, we transiently knocked down the expression of *GhDi19-3* and *GhDi19-4* in TRV: GhDi19-3 and TRV: GhDi19-4, respectively. After ten days of the VIGS induction, the positive control (TRV: GhCLA1) plants displayed an albino phenotype (Figure 6A). The transcript levels of *GhDi19-3* and *GhDi19-4* were found to be significantly decreased two weeks after VIGS (Figure 6B). This suggested that both *GhDi19-3* and *GhDi19-4* were successfully silenced in cotton plants.

Salt stress treatment was applied on the *GhDi19-3*- and *GhDi19-4*-silenced and control plants to evaluate the functions of *GhDi19-3* and *GhDi19-4* in salt stress response. No phenotypic differences were observed between the control and target gene-silenced (TRV: GhDi19-3, TRV: GhDi19-4) plants in mock treatment after ten days. However, the plants of the knock down of GhDi19-3 and GhDi19-4 showed a salt-insensitive phenotype under salt stress, while the control showed mild plant wilting and leaf yellowing phenotypes (Figure 6C). Plants accumulated excessive amounts of ROS (mainly O_2_^−^ and H_2_O_2_) in leaf cells when subjected to adverse environmental influences such as abiotic stress, causing oxidative damage to cells, disrupting cell membrane structure, and damaging plant cells and tissues. This ultimately affected plant metabolism and signal transduction [46]. MDA, POD, and SOD are known as the antioxidants that play important roles in stress responses and oxidative stress tolerance [47,48]. To investigate the regulation mechanism underlining the improved salt stress tolerance of the gene-silenced cotton plants, relevant physiological parameters, including H_2_O_2_, POD, MDA, and SOD contents, were analyzed in the target gene-silenced and control plants under different treatments (Figure 6D–G). Compared to the mock treatment, H_2_O_2_ contents in the leaves of the control plants were significantly induced under salt stress, while the H_2_O_2_ levels in leaves of TRV: GhDi19-3 and TRV: GhDi19-4 plants were significantly lower compared to the control (Figure 6D). Similar to the trend of H_2_O_2_, changes in leaves between mock treatment and salt treatment were also found in the comparison of POD activities. However, under mock and salt stress conditions, POD activities in the control plants were significantly higher than both gene-silenced (TRV: GhDi19-3, TRV: GhDi19-4) plants (Figure 6E). Under mock treatment, MDA levels in the gene-silenced (TRV: GhDi19-3, TRV: GhDi19-4) plants were significantly higher compared to the control. However, MDA levels increased after salt stress treatment, but were significantly lower compared to the control (Figure 6F). SOD activities in the gene-silenced (TRV: GhDi19-3, TRV: GhDi19-4) plants were higher compared to the control in both mock and salt stress treatment (Figure 6G). Together the results suggest that the improved salt stress tolerance of the *GhDi19-3* and *GhDi19-4* gene-silenced plants could be caused by the enhanced ROS-scavenging activity.

### 3.7. Expression Profiling of Calcium Signaling-Related and ABA-Responsive Genes in Simulated Salt Stress Treatment of Control and GhDi19-3- and GhDi19-4-Silenced Cotton Plants

Simulated salt stress treatment of the control and *GhDi19-3*- and *GhDi19-4*-silenced cotton plants revealed that the sensitivity to salt stress was decreased in *GhDi19-3*- and *GhDi19-4*-silenced cotton plants. To further explore the regulation mechanism to explain the phenotype of genes involved in calcium signaling and ABA signaling pathways, and based on previous studies [8,10,14,15,18,19,45,49] and the protein association network results, calcium signaling related genes (*GhCDPK2-1A*, *GhCDPK2-1D*, *GhCDPK2-2A*, *GhCDPK2-2D*, *GhCIPK11-A*, and *GhCIPK11-D*) and ABA-responsive genes (*GhABI5*, *GhRD29B-A*, and *GhRD29B-D*) expression profiling was analyzed to monitor ABA and calcium signaling pathways in cotton.

**Figure 7:**
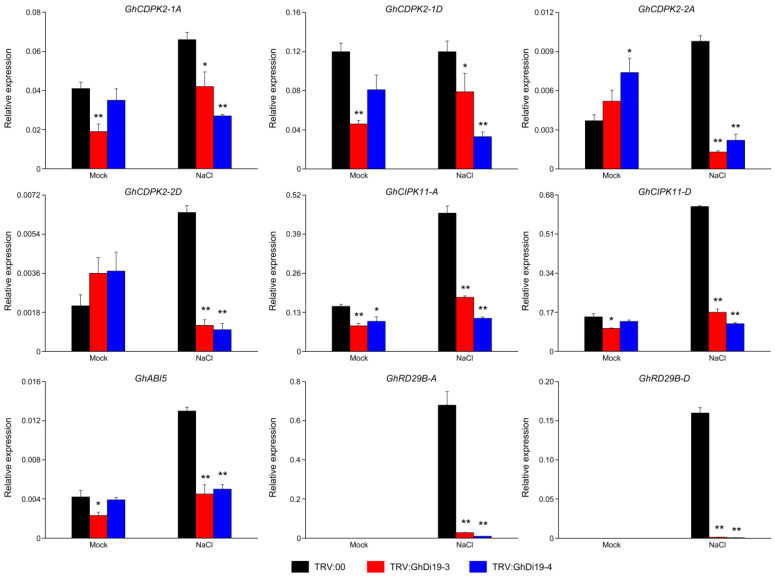
qRT-PCR analysis of calcium signaling related and ABA-responsive genes under simulated salt stress in the control and *GhDi19-3*- and *GhDi19-4*-silenced plants. Transcript levels of *GhCDPK2-1A*, *GhCDPK2-1D*, *GhCDPK2-2A*, *GhCDPK2-2D*, *GhCIPK11-A*, *GhCIPK11-D*, *GhABI5*, *GhRD29B-A*, and *GhRD29B-D* genes were determined by qRT-PCR, using *GhUBQ7* gene as the reference. Asterisks indicate significant differences (independent *t*-tests): * *p* < 0.05; ** *p* < 0.01.

Almost all genes were strongly induced in both the control and the *GhDi19-3* and *GhDi19-4* gene-silenced cotton plants under salt treatment after ten days (Figure 7). Transcript levels of four calcium-dependent protein kinase genes, including *GhCDPK2-1A*, *GhCDPK2-1D*, *GhCDPK2-2A*, and *GhCDPK2-2D*, were lower in *GhDi19-3*- and *GhDi19-4*-silenced cotton plants compared to the TRV: 00 plant under salt treatment. Under mock treatment, the expressions of *GhCDPK2-1A* and *GhCDPK2-1D* in the silenced plants were lower compared to the control, with a significant difference was observed only between *GhDi19-3*-silenced plants and the control. However, the expressions of *GhCDPK2-2A* and *GhCDPK2-2D* in the silenced cotton plants were higher compared to the control, where only *GhCDPK2-2A* expression showed a significant difference between *GhDi19-4*-silenced plants and the control. An exception was found in the expression of *GhCIPK11-D*, which had no significant difference between the *GhDi19-4*-silenced cotton plants and the control. The expressions of two *CIPKs*, *GhCIPK11-A* and *GhCIPK11-D*, in *GhDi19-3*- and *GhDi19-4*-silenced cotton plants before and after salt stress treatment were substantially lower compared to the control. Similarly, three ABA-responsive genes, including *GhABI5*, *GhRD29B-A*, and *GhRD29B-D*, expression levels were also substantially lower in *GhDi19-3*- and *GhDi19-4*-silenced cotton plants under salt treatment compared to the control. However, under mock treatment, it was surprising to observe that the mRNA levels of *GhRD29B-A* and *GhRD29B-D* were hardly detected between the control and *GhDi19-3*- and *GhDi19-4*-silenced cotton plants under mock treatment. Above results indicated that both GhDi19-3 and GhDi19-4, as two negative regulators, may participate in plant calcium signaling and ABA signaling pathways.

## 4. Discussion

Previous studies on the potential functions of Di19s in response to biotic or abiotic stress mainly focused on certain plant species [8,10,11,12,13,14,15,16,24]. However, these previous investigations were not extended to cotton species. In this study, we found that the Di19 protein was conserved in plants. Sixteen GhDi19 proteins were identified in upland cotton and clustered into five subgroups. All *GhDi19s* had a similar sequence structure which composed of five exons and four introns. Furthermore, all GhDi19s contained two zinc-finger domains and one NLS domain. Due to the presence of NLS, these Di19 proteins were localized in the nucleus. *GhDi19-3* and *GhDi19-4* genes encoding Di19 proteins were identified in cotton in addition to previously reported GhDi19-1 and GhDi19-2. Similar, to previously reported studies where GhDi19-1 and GhDi19-2 were found localized in the nucleus [10,26], subcellular localization of GhDi19-3 and GhDi19-4 suggested that both GhDi19 proteins were localized in the nucleus. These results laid the foundation to investigate the evolution of the *Di19s* in different species and further study the *Di19* gene family functions, especially in the pioneering of growing cotton in saline-alkali land.

Soil salinity is a major abiotic stress around the world, which seriously affects plant growth and productivity [1,50]. As salt-tolerant crop, corresponding stress response mechanisms in cotton have been widely investigated [50,51,52]. The transcriptome data show that both *GhDi19-3* and *GhDi19-4* have similar expression dynamics to the previously reported results of *GhDi19-1* and *GhDi19-2* under salt stress, indicating potential functions in salt stress response. Abiotic stress-related cis-elements, including MBS, STRE, MYC, etc., were identified in *GhDi19-3* and *GhDi19-4* promoter regions. It was speculated that the sensitivity of *GhDi19-3* and *GhDi19-4* to salt stress may be regulated by abiotic stress-related cis-elements in these gene’s upstream promoter. Interestingly, the cotton plants with a reduction of *GhDi19-3* and *GhDi19-4* expression by the VIGS system have accelerated the tolerance to salt stress. Hence, *GhDi19-3* and *GhDi19-4* are two candidate genes for enhancing the salt-tolerance of cotton in the future.

**Figure 8:**
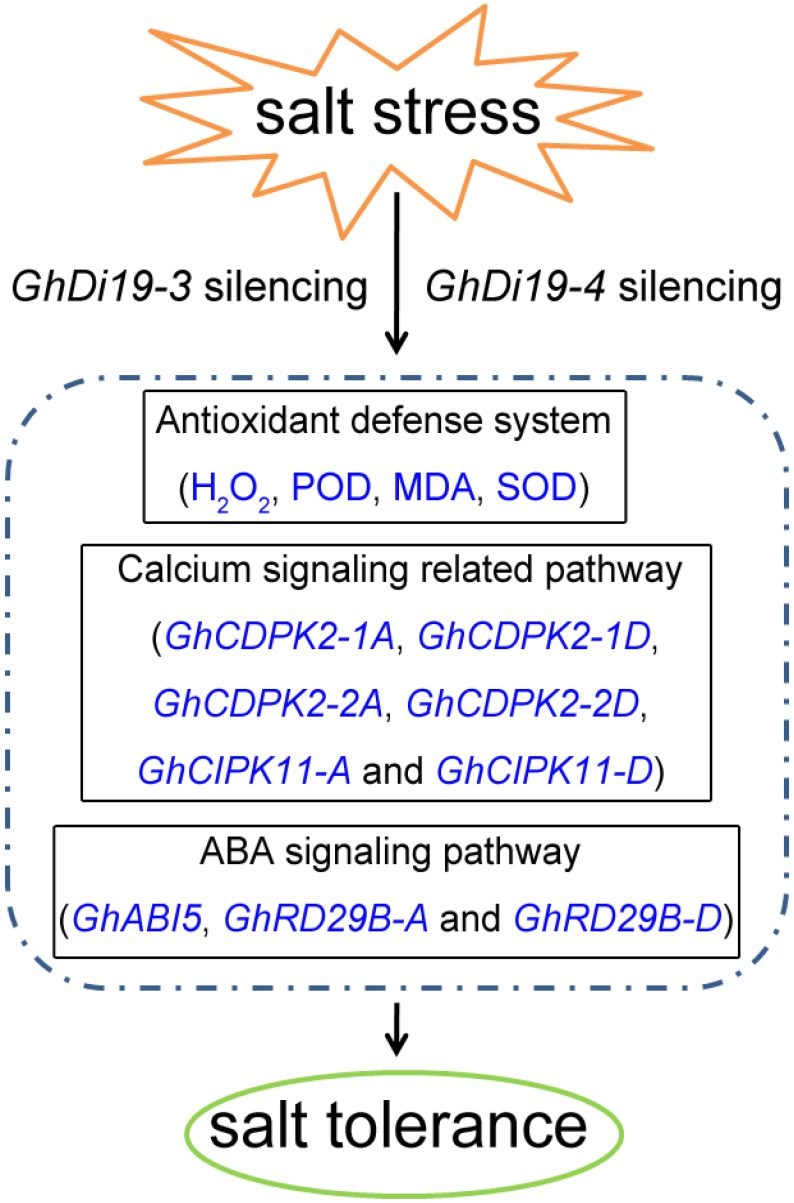
A model describing the silenced *GhDi19-3* and *GhDi19-4* gene regulates cotton tolerance to salt stress. *GhDi19-3* and *GhDi19-4* were up-regulated under salt stress, and the silencing of *GhDi19-3* or *GhDi19-4* genes led to a change in up- and down-regulation of calcium signaling and ABA signaling pathway related genes (*GhCDPK2-1A*, *GhCDPK2-1D*, *GhCDPK2-2A*, *GhCDPK2-2D*, *GhCIPK11-A*, *GhCIPK11-D*, *GhABI5*, *GhRD29B-A*, and *GhRD29B-D*), which then resulted in their abilities improving to effectively reduce the ROS damage when cotton plants were subject to salt stress. Taken together, GhDi19-3 and GhDi19-4, as two negative regulatory transcription factors, responded to salt stress by being involved in calcium signaling and ABA signaling pathways.

Previous studies have demonstrated that AtDi19-3 was involved in plant responses to drought and high salt stress conditions [16] and functions as a negative regulator under drought stress by interacting with AtCIPK11 [19]. In addition, AtDi19-3 interacts with IAA14 in the auxin signal transduction [20], indicating that AtD19-3 have important functions in regulating plant responses to stress as well as to hormone signals. In this study, bioinformatics analysis indicated that GhDi19-3 and GhDi19-4 have the highest identify to the At19-3 protein of the *AtDi19* gene family, and we speculate that GhDi19-3, GhDi19-4, and AtDi19-3 had the same function in response to stress. *AtDi19-3* expression was induced under salt stress, and its mutant exhibits enhanced salt tolerance [14,16]. In our study, results of the simulated salt stress treatment of the control and *GhDi19-3*- and *GhDi19-4*-silenced cotton plants, similar to the phenotypic changes of *Atdi19-3*, showed a decreased sensitivity to salt stress. This suggested that *GhDi19-3* and *GhDi19-4* are negative regulators in salt stress response. Further, it suggests that GhDi19-1 and GhDi19-2 are from the same evolutionary branch as GhDi19-3 and GhDi19-4, which through phosphorylation of serine residue regulate the stress response of cotton through mediating the ABA signal transduction [10,26]. The results of physiological parameters measured in the simulated salt stress treatment of the control and *GhDi19-3*- and *GhDi19-4*-silenced cotton plants showed that silencing of *GhDi19-3* and *GhDi19-4* genes regulated the scavenging ability of ROS, thereby enhancing plants tolerance to salt stress. The expression patterns of the proteins regulatory pathways and their related genes in the simulated salt stress treatment of the control and *GhDi19-3*- and *GhDi19-4*-silenced cotton plants further showed that certain calcium signaling and ABA-responsive genes are up- and down-regulated under salt stress treatment. Both *GhDi19-3* and *GhDi19-4* may participate in calcium signaling and ABA signaling pathways in response to salt stress. Additionally, we speculate that GhDi19-3 and GhDi19-4 may have redundant functions similar to *GhDi19-1* and *GhDi19-2*, which need to be verified in the future. Based on the above results, our study provided a model to visually describe the elucidated mechanisms of *GhDi19*-3 and *GhDi19-4* genes in response to salt stress (Figure 8). GhDi19-3 and GhDi19-4, as two negative regulatory transcription factors, respond to salt stress through calcium signaling and ABA signaling pathways.

## 5. Conclusions

In this study, 82 Di19 proteins have been identified from 10 plant species, and the characteristics of the *GhDi19* gene family from three cotton species, including the analysis of the gene number, gene structure, chromosomal distribution, promoter cis-elements, and expression profiles have been systematically investigated and analyzed. *GhDi19* members have shared similar conserved structures of five exons and four introns. The GhDi19 proteins have five highly conserved motifs. All GhDi19 proteins contain the Di19_zinc-binding domain, the Di19_C terminal domain, and a nuclear localization signal. Moreover, the cis-elements related to abiotic stress such as ARE, MYC, and STRE have been found at the promoter of *Di19* genes. Transcriptomic analysis of *Di19* genes in different tissues and their responses to abiotic stresses indicate that certain *GhDi19s* may play a significant role in the growth and development of upland cotton. Among them, *GhDi19-3* and *GhDi19-4* are up-regulated under salt stress. GhDi19-3 and GhDi19-4 are used as candidate proteins to further verify their potential roles in salt stress response. Subcellular localization indicates that the two GhDi19s are nuclear-localized proteins. *GhDi19-3* and *GhDi19-4* have decreased the sensitivity under salt stress through VIGS, and have showed significantly lower levels of H_2_O_2_, MDA, and POD as well as significantly increased SOD activity, this suggests that the tolerance to salt stress was improved by reducing the ROS damage. Further, certain calcium signaling and ABA-responsive gene expression levels shows up- and down-regulation changes in target gene-silenced plants, suggesting that *GhDi19-3* and *GhDi19-4* may be involved in calcium signaling and ABA signaling pathways in response to salt stress. Overall, our findings indicate that GhDi19-3 and GhDi19-4, as two negative regulatory transcription factors, respond to salt stress by being involved in calcium signaling and ABA signaling pathways. The comprehensive understanding of the physiological and molecular mechanisms of *GhDi19-3* and *GhDi19-4* can serve as an important genetic resource for the improvement in salinity stress tolerance and the yield of cotton.

## Data Availability

The data presented in this study are available in the Supplementary Materials.

## Supplementary Materials

The online version contains Supplementary Material available at: https://www.mdpi.com/article/10.3390/antiox11112225/s1, Figure S1. Chromosomal localization of *Di19s* on *G. arboreum* (A), *G. raimondii* (B) and *G. hirsutum* (C); Figure S2. Expression profile of *GhDi19s* in different tissues of *G. hirsutum*. Table S1, The primers used in this study. Table S2, Information of *Di19s* in different plant species. Table S3, Analysis conserved cis-elements of *GhDi19* gene promoters. Table S4, The transcriptomic data analysis of *GhDi19s* in different stresses. Table S5, The transcriptomic data analysis of *GhDi19s* in different tissues.
