# Supplementary figures and images for: Systematical Characterization of the Cotton Di19 Gene Family and the Role of GhDi19-3 and GhDi19-4 as Two Negative Regulators in Response to Salt Stress
Source: Antioxidants (Basel). 2022 Nov 11;11(11):2225. doi: 10.3390/antiox11112225 (PMC9686973; doi:10.3390/antiox11112225)

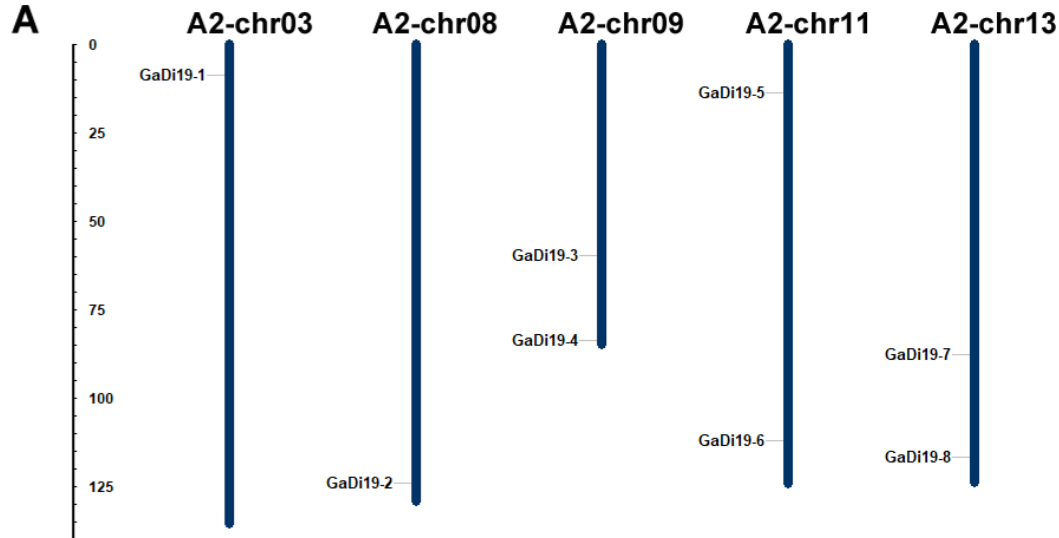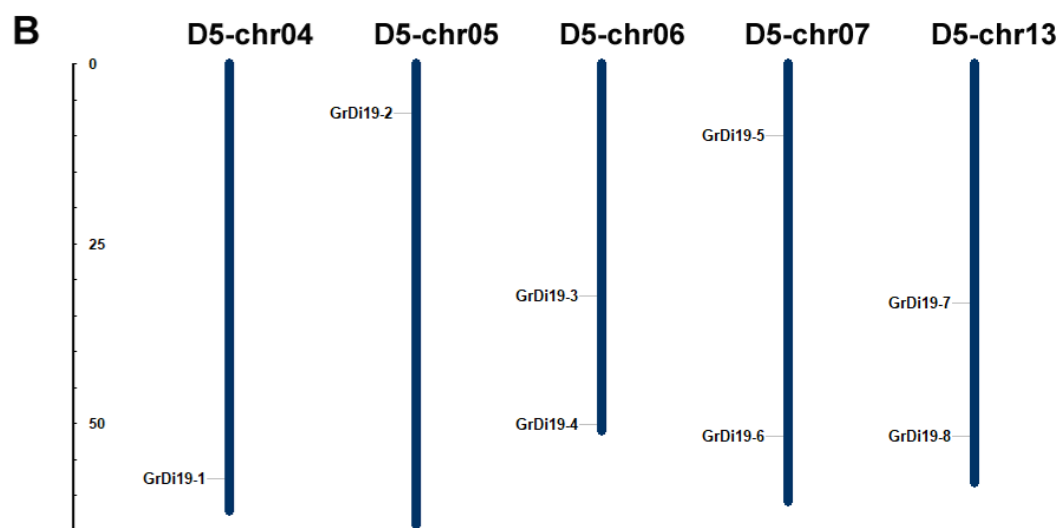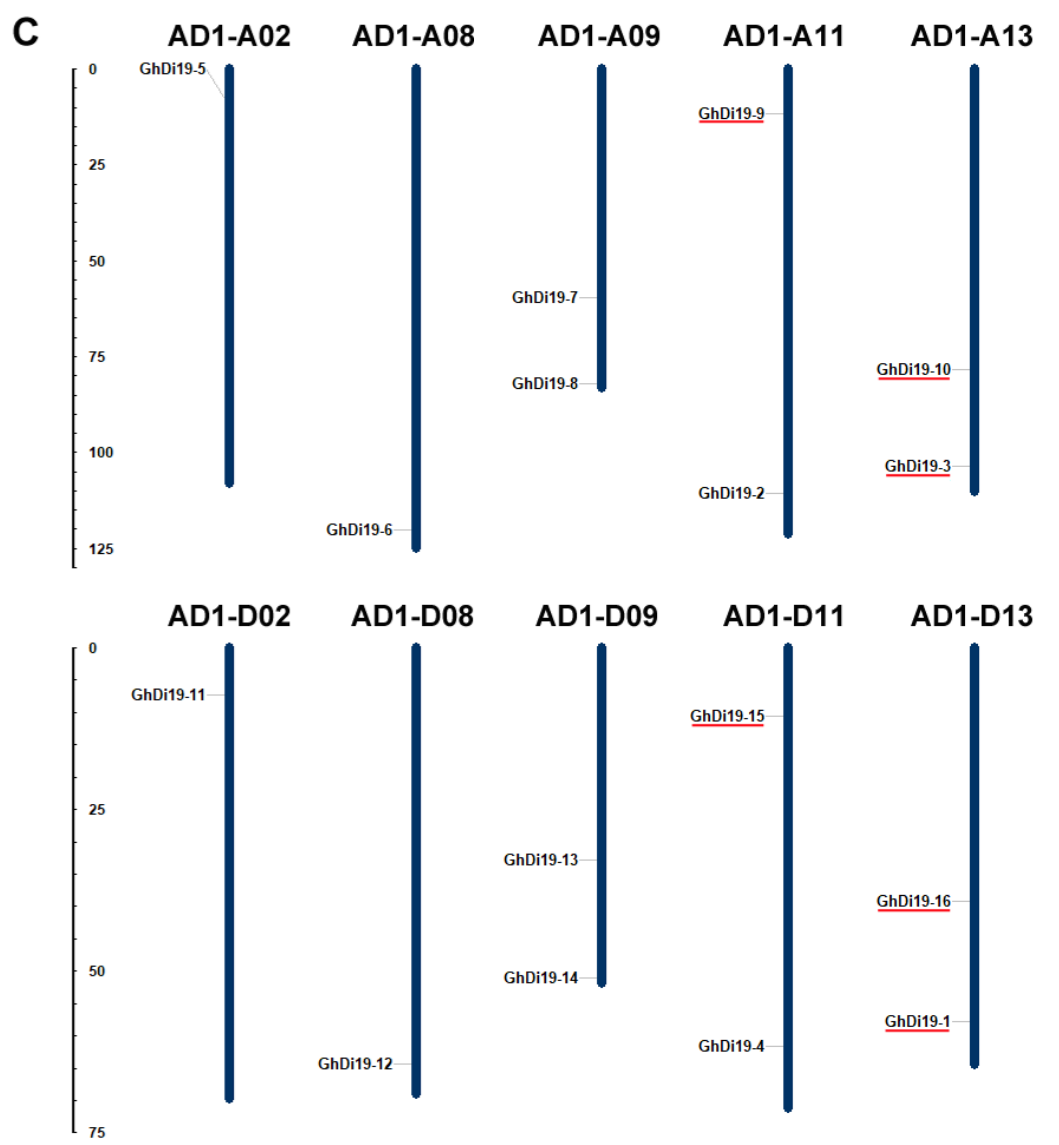

Supplement: Supplementary file 1 [file antioxidants-11-02225-s001.zip › Supplementary Figure S1.pdf]

A

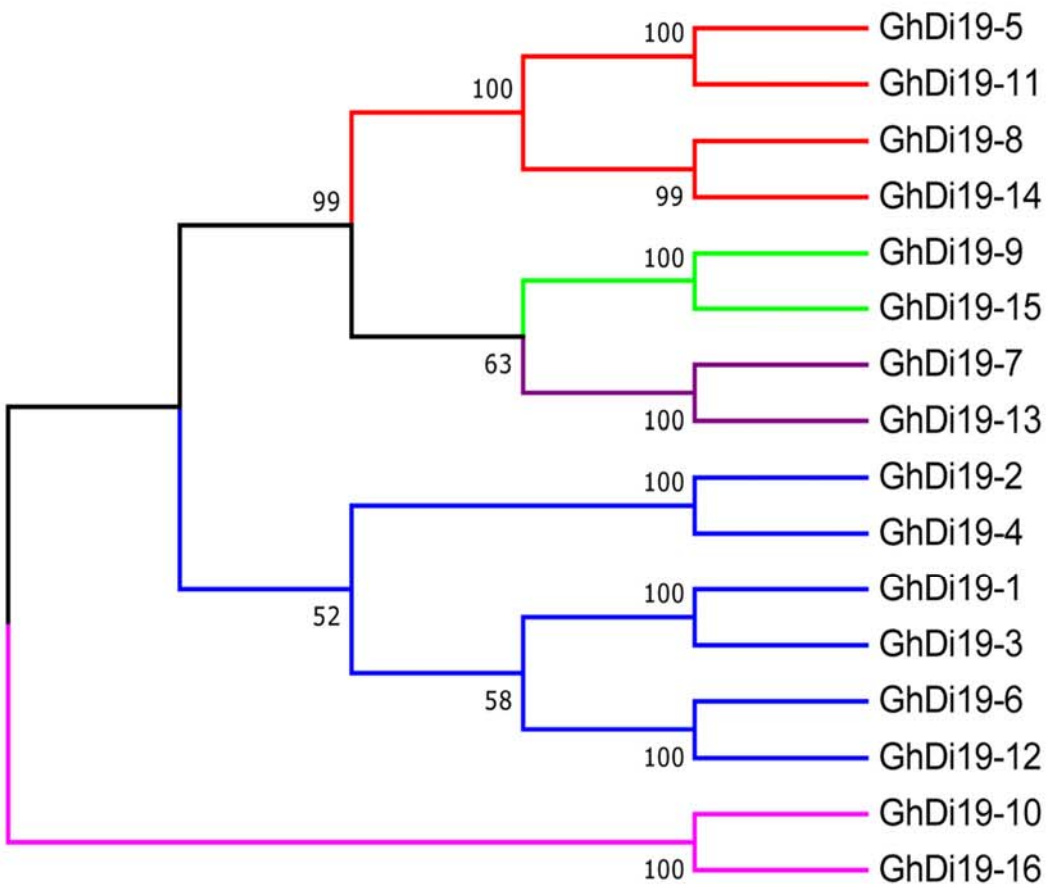

B

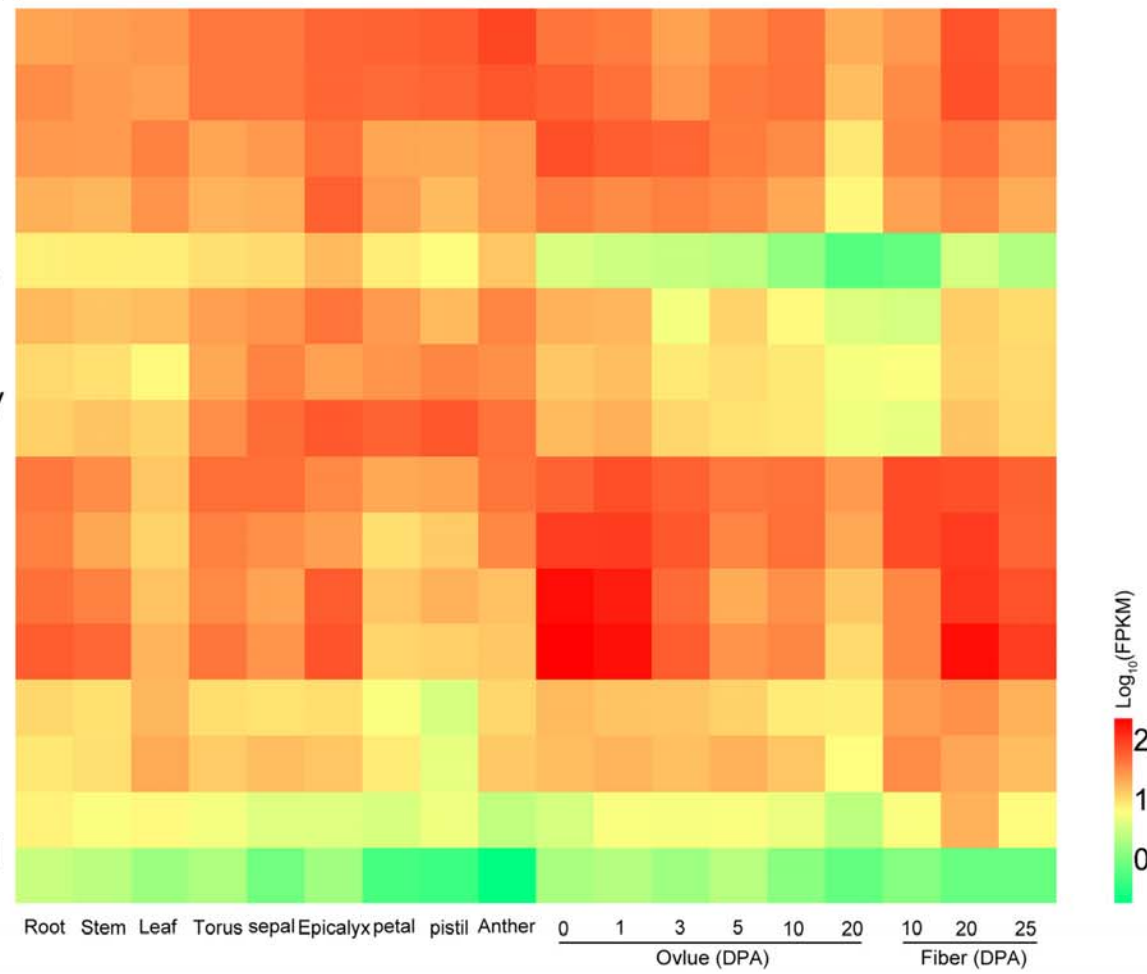

Supplement: Supplementary file 1 [file antioxidants-11-02225-s001.zip › Supplementary Figure S2.pdf]
